# Store and neighborhood differences in retailer compliance with a local staple foods ordinance

**DOI:** 10.1186/s12889-020-8174-2

**Published:** 2020-02-04

**Authors:** Caitlin E. Caspi, Megan R. Winkler, Kathleen M. Lenk, Lisa J. Harnack, Darin J. Erickson, Melissa N. Laska

**Affiliations:** 1 Department of Family Medicine and Community Health, Program in Health Disparities Research, University of Minnesota, 717 Delaware St. SE, Minneapolis, MN USA; 2 Division of Epidemiology and Community Health, Suite 300, University of Minnesota, 1300 South 2nd St, Minneapolis, MN USA

**Keywords:** Food access, Policy, Small food store, Stocking standards, Neighborhood disparities

## Abstract

**Background:**

Policies to improve healthy food retail have been recognized as a potential means of reducing diet-related health disparities. The revised 2014 Minneapolis Staple Foods Ordinance instituted minimum stocking standards for healthy, staple foods. The objective of this study was to examine retailer compliance with the policy, and whether compliance varied by neighborhood and store characteristics.

**Methods:**

In this natural experiment, audits were conducted annually pre- and post-ordinance (2014–2017) in 155 small/nontraditional stores in Minneapolis, MN and a comparison city (St. Paul, MN). Compliance measures for 10 product categories included: (1) met requirements for ≥8 categories; (2) 10-point scale (one point for each requirement met); and (3) carried any item in each category. Store characteristics included store size and ownership status. Neighborhood characteristics included census-tract socioeconomic status and low-income/low-access status. Analyses were conducted in 2018.

**Results:**

All compliance measures increased in both Minneapolis and St. Paul from pre- to post-policy; Minneapolis increases were greater only for carrying any item in each category (*p* < 0.01). In Minneapolis, corporate (vs. independent) stores were generally more compliant. No differences were found by neighborhood characteristics.

**Conclusions:**

Overall trends suggest broad movement among Minneapolis stores towards providing a minimum level of staple foods. Increases were greater in corporate stores. Trends do not suggest neighborhood-level disparities in compliance.

**Study registration:**

ClinicalTrials.gov NCT02774330, retrospectively registered May 17, 2016.

## Background

Disparities in the healthfulness of local food environments are evident across the U.S. [1, 2] High-minority and low-income neighborhoods are less likely to have supermarkets and more likely to have small food stores [1–3]. Small and non-traditional stores, such as corner stores, gas-marts, dollar stores, and pharmacies, have consistently demonstrated a limited selection of healthy food items [4–6], with managers of these stores citing challenges in procuring, stocking, and selling health food [7–10]. Unequal access to healthy food is associated with disparities in diet-related health outcomes, including obesity [11–15].

Policies to improve healthy food retail have been recognized as a potential means of reducing diet-related health disparities [16]. Some policies, such as the 2010 Healthy Food Financing Initiative, focus on geographic areas defined by the U.S. Department of Agriculture (USDA) as low-income areas with low access to healthy food [17, 18]. Other types of retailer policies are not focused on particular geographic areas, but aim to increase healthy food in retail settings that serve low-income groups at risk of poor diet, such as the 2009 revision of retailer standards for the Special Supplemental Nutrition Program for Women, Infants and Children (WIC) [19–21]. The WIC policy change resulted in improved availability and variety of healthy foods in WIC-authorized stores [19–21]. A similar proposed policy strategy at the local level is creating minimum stocking requirements for healthy foods in all types of food stores as a condition of business licensing [22].

In 2008, the City Council of Minneapolis, MN passed the first Staple Foods Ordinance in the U.S., requiring licensed grocery stores to carry a basic minimum of healthy, staple foods. In 2014, the ordinance was revised to better align the policy with the Dietary Guidelines for Americans and stocking requirements for retailers participating in WIC [23, 24]. Revisions established minimum stocking requirements in 10 product categories, including fruits and vegetables, whole grain rich products, and low-fat dairy. The revised Staple Foods Ordinance had few exemptions; it applied not only to traditional large and medium-sized grocery stores, but also smaller non-traditional stores (such as gas-marts, dollar stores, and pharmacies) that sold food and participated in government food assistance programs like the Supplemental Nutrition Assistance Program (SNAP). The intention of the ordinance was to address disparities in food access within the city, recognizing the lack of universal access to supermarkets and the need to “ensure that everyone has access to healthy foods no matter where they shop” [25].

The revised Staple Foods Ordinance was first implemented in April 2015 with a one-year period without enforcement to address compliance concerns from retailers. During this year, the Minneapolis Health Department implemented the policy by assessing retailers’ understanding of policy requirements, conducting training on product procurement/marketing, offering resources like merchandising kits for infrastructure enhancements, and conducting meetings with corporate chain store representatives. Enforcement began in 2016 and was carried out through routine health inspections in which inspectors were given the authority to issue warning letters, citations, and fines for non-compliance.

An evaluation of the Minneapolis Staple Foods Ordinance in small and non-traditional stores revealed challenges with the implementation of the ordinance [26]. By 2017, only 9.6% of small and non-traditional food stores were fully compliant with the requirements, and changes in compliance during the study period (2014–2017) were not statistically different from analogous changes in inventory among stores in the comparison city (St. Paul, MN). However, while not statistically different from St. Paul, stores in Minneapolis showed significant improvements in compliance and healthy food availability over the study period. Looking at more detailed changes in store stocking using difference compliance indicators can contribute a better understanding of implementation successes and challenges.

The ability of stores to comply with the Staple Foods Ordinance could be affected by store and neighborhood factors that influence the ability to stock healthy food. Research has shown that small food stores in low-income/low-access neighborhoods have dense networks of unhealthy food suppliers [27], and major food distributors may avoid deliveries to smaller and independently-owned stores [7, 10]. The smallest stores tend to stock less healthy food than even slightly larger ones, perhaps due to difficulty in healthy food procurement among the smallest stores [4]. Stores with fewer supports for supplying healthy food could be less likely to comply with the policy, resulting in an unintended exacerbation of disparities in healthy food access.

Given the trends toward compliance noted in our previous study, the objectives of this study were to: 1) assess three distinct indicators of movement towards compliance, described in detail below, and 2) assess compliance in different stores and neighborhoods. We hypothesized that larger and corporately-owned stores (versus smaller and independently-owned stores) would demonstrate larger increases in compliance from baseline, as would stores located in higher-SES or outside low-income/low-access areas (versus those in lower-SES areas or in low-income/low-access areas).

## Methods

### Study Design

This natural experiment involved 4 annual time points of data collection (T1-T4) from 2014 to 2017 in stores in Minneapolis, MN (where the Staple Foods Ordinance was being implemented) and St. Paul, MN (the comparison community).

### Study sample

The study sample and store recruitment process has been described previously [5, 6, 26, 28–33]. Stores exempted from the ordinance (including those that would not reasonably be expected to stock a minimal amount of foods and stores located the core downtown commercial district) were excluded from the evaluation in Minneapolis and St. Paul. The evaluation study targeted retailers that were not already expected to meet the new minimum stocking requirements; thus supermarkets, mass-merchandizers, and stores participating in the Special Supplemental Nutrition Program for Women, Infants, and Children (WIC) were excluded from the sample. Finally, stores with invalid licensing addresses were excluded.

Of 255 eligible stores, 180 were randomly selected to participate. After visiting these stores to collect data at baseline (T1), 23 were identified as ineligible (e.g., due to new participation in WIC), and an additional 17 refused to participate. At the three subsequent data collection time points, study staff re-visited stores that refused to participate at T1 to attempt data collection. The final analytic sample had 155 unique stores (*n* = 140 at T1, 139 at T2, 137 at T3, and 127 at T4). Among the eligible sample, all stores were categorized as corner stores, gas-marts, dollar stores, pharmacies, or general retailers at T1. The study was approved by the Institutional Review Board at University of Minnesota.

### Measures

Store environment was assessed using a tool modified from the Rudd Center for Food Policy and Obesity that was developed to evaluate changes in WIC policy revisions in small food stores in 2009 [20]. The tool, described previously [5, 6, 26, 28], is similar in format to Nutrition Environment Measure Survey in Stores (NEMS-S), but modified to align with the 10 Minneapolis Staple Food Ordinance requirements.

From the data generated using the store assessment tool, three indicators of ordinance compliance were generated:

1. *80% compliance with ordinance requirements*: the percent of stores that met at least 8/10 of the product category requirements of the ordinance (yes/no).
2. *10-point scale:* the total number of ordinance requirements met by stores (range 0–10), presented as an average across stores.
3. *Carrying any food in each of the 10 categories:* whether stores had *any* food in each of the categories required by the ordinance, even if the food was not in the appropriate package size, form, or quantity required by the ordinance (e.g., eggs sold in dozen containers were required by the ordinance, but eggs packed in half-dozen containers met the criteria for *any eggs)*. The indicator is presented as the percent of stores that had any in all 10 categories (yes/no).

Store size (small/larger) was measured during the assessment as the number of cash registers in the store. Small stores had 1–2 cash registers and larger stores had at least 3 cash registers.

Store ownership status (corporate/independent) was determined during an interviewer-administered survey with store managers in which they were asked whether the store was independently-owned, corporately-owned, or part of a franchise. Franchise and corporately-owned stores were combined into a single category. In stores where ownership status was not available from the manager survey, two study team members (CEC and MNL) determined status based on publicly available information about the store (e.g., name, number of locations). Stores that were part of well-known chains were assigned corporate status; stores that had only one location were deemed independent.

Neighborhood data were obtained from 5-year American Community Survey estimates (ACS, 2009–2015) [34] and attributed to stores based on census tract location. Store census tracts were classified into lower-socioeconomic status (SES) or higher-SES. Lower-SES census tracts had > 50% of residents at or below 185% of the federal poverty income guidelines [35].

Using the USDA Food Access Research Atlas [17], stores were classified as low-income/low-access if the census tract they were located in was both low-income and low-access. Low-income tracts met any of the following criteria: [1] median family income ≤80% of the state-wide of the metropolitan area’s median family income [2]; poverty rate > 20%. Low-access tracts had ≥100 households located > 1/2 mile from the nearest supermarket and had no access to a vehicle.

### Statistical analyses

Descriptive statistics were computed for store and neighborhood characteristics at baseline for Minneapolis and St. Paul separately, expressed as number and percentage of stores/neighborhoods. We also computed chi-square tests to compare the store and neighborhood characteristics across the two cities. Using data from T1-T4 from both cities, a mixed regression model for each of the three compliance outcome measures was conducted to examine the overall movement towards compliance in Minneapolis compared with the control condition in St. Paul. For each model, we tested an overall time-by-city interaction, adjusted for neighborhood race/ethnicity (the only significant covariate in bivariate city comparisons).

Subsequent analyses were limited to stores in Minneapolis only, to compare magnitude of changes in compliance within different stores and neighborhoods of the policy area. A mixed regression model was computed for each compliance outcome with store (size, ownership status) and neighborhood characteristics (SES, low-income/low-access status) as independent variables. Models tested the interaction between time and store/neighborhood characteristic (3 degrees of freedom) for each outcome. For interactions that reached statistical significance, we tested changes between baseline and each time point (T1 to T2, T1 to T3, and T1 to T4). All models were adjusted for repeated measures over time. All analyses were conducted in SAS (SAS/STAT Version 9.4).

## Results

Table 1 presents store characteristics in Minneapolis and St. Paul. Most stores were corner/convenience stores or food-gas marts (75% in Minneapolis vs. 73% in St. Paul). The remainder were dollar stores, pharmacies, and one general retailer in Minneapolis. The majority of stores in both cities were smaller, located in higher SES neighborhoods, and located outside of low-income/low-access neighborhoods. There were more independent stores in Minneapolis (55%) vs. St. Paul (41%). Correlations between store and neighborhood characteristics were small, except store size and ownership status was moderately correlated (r = 0.6).

**Table 1:** Store and neighborhood characteristics at baseline, Minneapolis and St. Paul, MN, 2014

Store and neighborhood characteristics	Minneapolis *(n* = 77)	St. Paul *(n* = 63)	
	*n (%)*	*n (%)*	*p-value* ^*a*^
Store type			0.49
Corner stores, convenience stores, small groceries	34 (44)	20 (32)	
Food-gas marts	24 (31)	26 (41)	
Dollar stores	7 (9)	6 (10)	
Pharmacies	11 (14)	11 (17)	
General retailers	1 (1)	0 (0)	
Store size			0.39
Smaller size (1–2 registers)	53 (69)	39 (62)	
Larger size (3+ registers)	24 (31)	24 (38)	
Store ownership status			0.12
Corporate	35 (45)	37 (59)	
Independent	42 (55)	26 (41)	
Neighborhood SES			0.28
Lower SES (< 185% of poverty)	26 (34)	16 (25)	
Higher SES (> = 185% of poverty)	51 (66)	47 (75)	
Neighborhood low-income/low-access status			0.11
Store located in low-income/low-access area	23 (30)	27 (43)	
Store not located in low-income/low-access area	54 (70)	36 (57)	

^a^ Chi-square tests

Table 2 presents the results of models for the three compliance indicators by city, as well as change in these measures over the four time points. All indicators were higher in Minneapolis than St. Paul at every time point, and all indicators demonstrated a net increase between T1 to T4 in both cities. Changes were only statistically significant for the percent of stores that carried any food in each of the 10 categories (*p* = 0.01) in Minneapolis (27.6 to 75.1%), compared to St. Paul (12.3 to 19.1%).

**Table 2:** Compliance in Minneapolis vs. St. Paul

Compliance Outcome	City	Pre-policy (2014) Mean (SE)	Implementation only (2015) Mean (SE)	Early initiation of enforcement (2016) Mean (SE)	Continued monitoring (2017) Mean (SE)	Overall Effect (time-by-city) *P* (df = 3)
Met 80% of ordinance standards (% of stores)	Minneapolis (Policy)	24.4 (4.9)	40.7 (5.6)	31.0 (5.2)	50.5 (5.9)	0.83
	St. Paul (Control)	3.2 (2.2)	11.6 (4.1)	6.7 (3.2)	14.4 (4.7)	
10 point scale (0–10; 1 point for each standard met)	Minneapolis (Policy)	5.7 (0.3)	6.4 (0.3)	6.6 (0.2)	7.1 (0.2)	0.30
	St. Paul (Control)	4.4 (0.2)	4.7 (0.3)	4.9 (0.3)	5.3 (0.3)	
Have any food in each of 10 categories (% of stores)	Minneapolis (Policy)	27.6 (5.2)	39.0 (5.5)	51.1 (5.8)	75.1 (5.1)	0.01
	St. Paul (Control)	12.3 (4.1)	12.7 (4.2)	16.3 (4.7)	19.1 (5.2)	
	*p*-net		*p* = 0.27	*p* = 0.13	*p* = 0.0009	

Note: Regression models are adjusted for repeated measures over time and for neighborhood race/ethnicity (the only covariate that was significant in bivariate comparisons between Minneapolis and St. Paul); *p*-net values refer to changes in time-by-city effect from pre-policy to implementation only, from pre-policy to early initiation of enforcement, from pre-policy to continued monitoring respectively bolded values: *p* ≤ .05

**Fig. 1:**
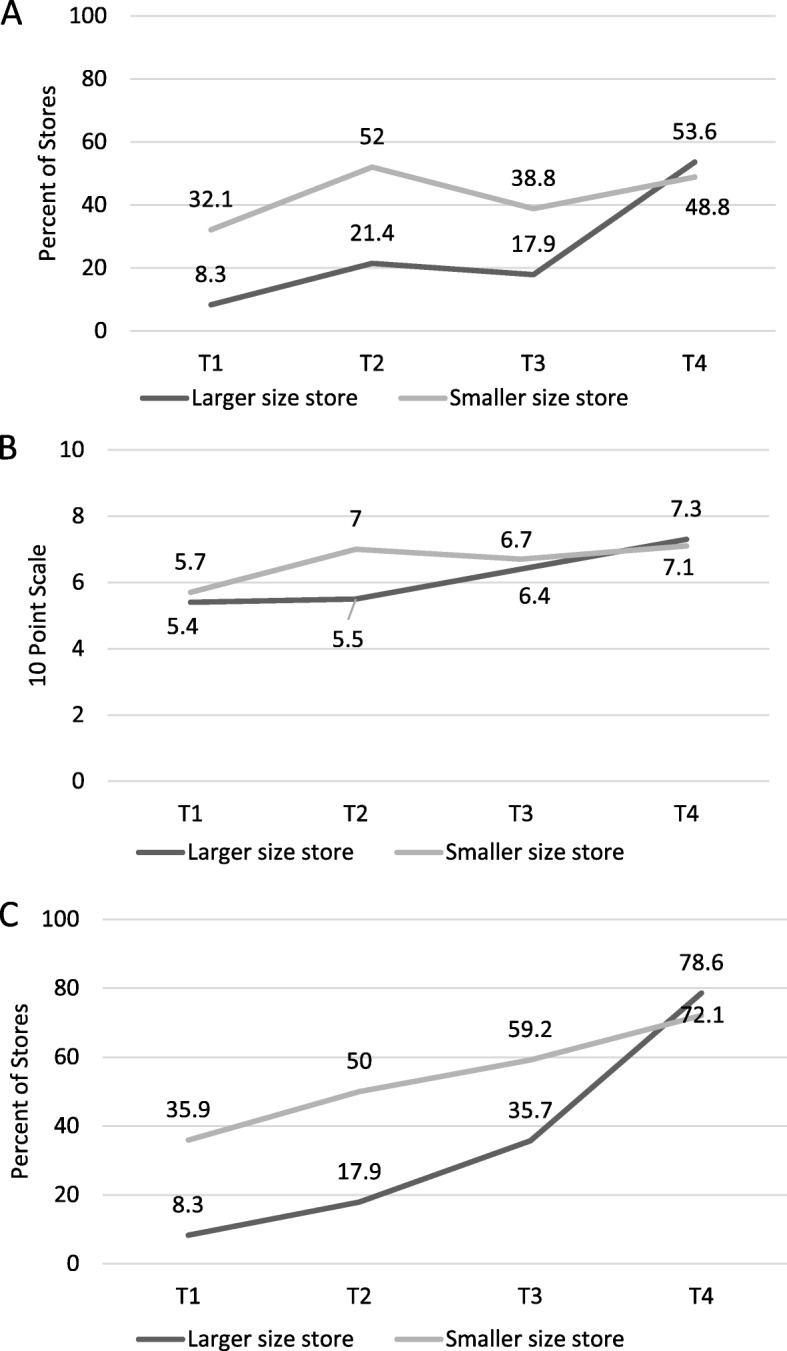
Compliance with the Minneapolis Staple Foods Ordinance in Larger and Smaller Stores in Minneapolis, MN **A**: Percent of Stores Meeting 80% of Staple Foods Ordinance Standards; **B**: 10-Point Scale of Meeting Ordinance Requirements; **C**: Percent of Stores Stocking Any Food in All 10 Categories; T1 = 2014 (Pre-policy), T2 = 2015 (Implementation only), T3 = 2016 (Early initiation of enforcement), T4 = 2017 (Continued monitoring).

**Fig. 2:**
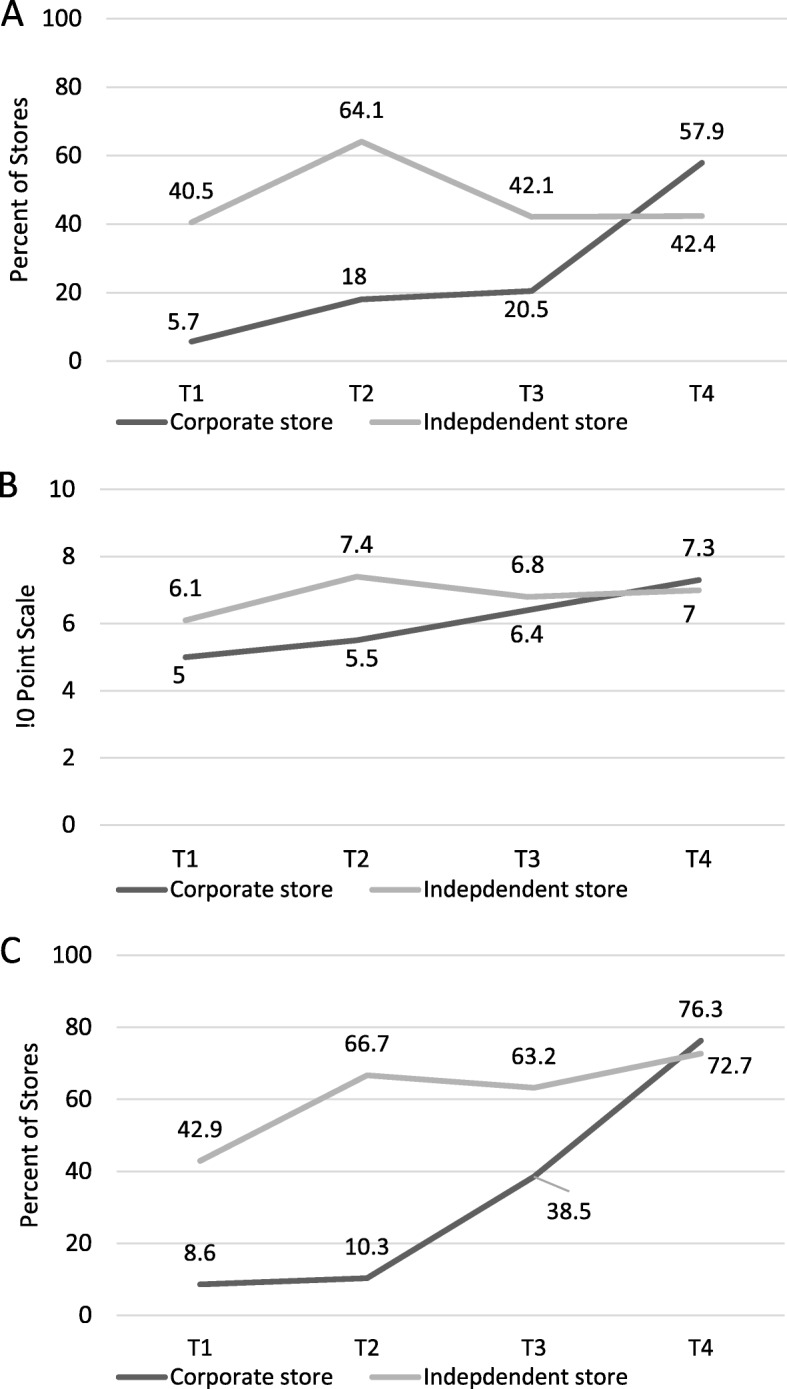
Compliance with the Minneapolis Staple Foods Ordinance in Corporate and Independent Stores in Minneapolis, MN **A**: Percent of Stores Meeting 80% of Staple Foods Ordinance Standards; **B**: 10-Point Scale of Meeting Ordinance Requirements; **C**: Percent of Stores Stocking Any Food in All 10 Categories; T1 = 2014 (Pre-policy), T2 = 2015 (Implementation only), T3 = 2016 (Early initiation of enforcement), T4 = 2017 (Continued monitoring)

Table 3 presents the results of models and significance testing for the three compliance indicators in Minneapolis alone by store size and ownership status; Figs. 1 and 2 visually present trends in compliance by these store factors over time. Compared with smaller-sized stores, larger stores had lower compliance on all indicators at T1, but by T4 surpassed smaller-sized stores on every indicator. However this increase was only statistically significant for the 10 point scale (*p* = 0.03), for which smaller stores initially had a greater gain from T1 to T2 than larger stores, but this gain was subsequently surpassed by larger stores at T4. Corporate stores (versus independent stores) had lower compliance for all indicators at baseline, but had significantly surpassed independent stores at T4 for all indicators.

**Table 3:** Compliance by store characteristics (Minneapolis only)

Compliance Outcome	Store characteristic	Pre-policy (2014) Mean (SE)	Implementation only (2015) Mean (SE)	Early initiation of enforcement (2016) Mean (SE)	Continued monitoring (2017) Mean (SE)	Overall Effect (time-by-characteristic) *p* (df = 3)
Met at least 8 of 10 ordinance standards (% of stores)	Larger size (3+ registers)	8.3 (5.6)	21.4 (7.8)	17.9 (7.2)	53.6 (9.4)	0.08
	Smaller size (1–2 registers)	32.1 (6.4)	52.0 (7.1)	38.8 (7.0)	48.8 (7.6)	
	Corporate	5.7 (3.9)	18.0 (6.1)	20.5 (6.5)	57.9 (8.0)	<.0001
	Independent	40.5 (7.6)	64.1 (7.7)	42.1 (8.0)	42.4 (8.6)	
	*p*-net		0.66	0.09	0.0002	
10 point scale (0–10; 1 point for each category where standard met)	Larger size (3+ registers)	5.4 (0.3)	5.5 (0.4)	6.4 (0.3)	7.3 (0.3)	0.03
	Smaller size (1–2 registers)	5.7 (0.4)	7.0 (0.4)	6.7 (0.3)	7.1 (0.3)	
	p-net		0.03	0.94	0.43	
	Corporate	5.0 (0.3)	5.5 (0.3)	6.4 (0.3)	7.3 (0.3)	0.0008
	Independent	6.1 (0.5)	7.4 (0.4)	6.8 (0.4)	7.0 (0.4)	
	*p*-net		0.08	0.16	0.01	
Have any food in each of 10 categories (% of stores)	Larger size (3+ registers)	8.3 (5.6)	17.9 (7.2)	35.7 (9.1)	78.6 (7.8)	0.08
	Smaller size (1–2 registers)	35.9 (6.6)	50.0 (7.1)	59.2 (7.0)	72.1 (6.8)	
	Corporate	8.6 (4.7)	10.3 (4.9)	38.5 (7.8)	76.3 (6.9)	0.002
	Independent	42.9 (7.6)	66.7 (7.5)	63.2 (7.8)	72.7 (7.8)	
	*p*-net		0.25	0.12	0.006	

Note: All models adjusted for repeated measures over time; *p*-net values refer to changes in time-by-characteristic effect from pre-policy to implementation only, from pre-policy to early initiation of enforcement, from pre-policy to continued monitoring respectively; bolded values: *p* ≤ .05

**Fig. 3:**
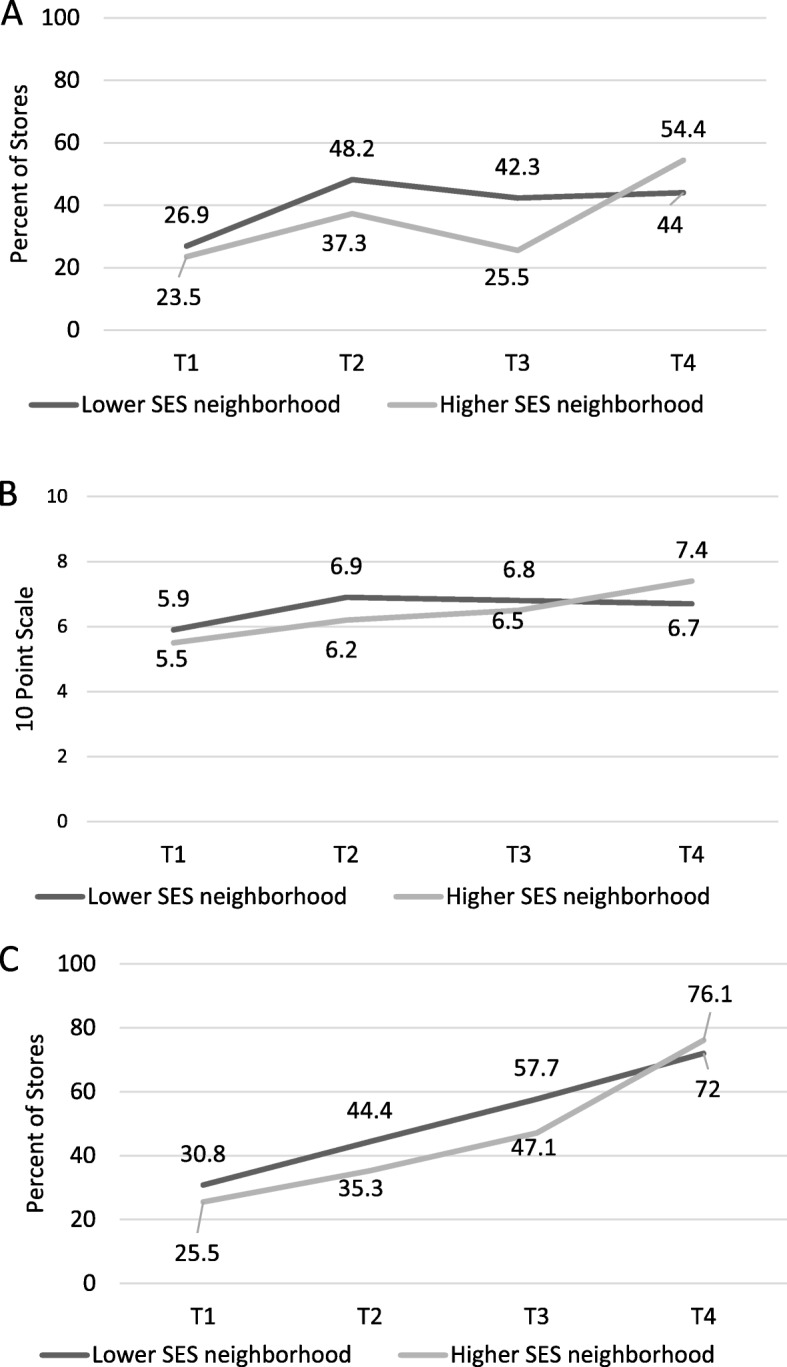
Compliance with the Minneapolis Staple Foods Ordinance in Lower-SES and Higher-SES Stores in Minneapolis, MN **A**: Percent of Stores Meeting 80% of Staple Foods Ordinance Standards; **B**: 10-Point Scale of Meeting Ordinance Requirements; **C**: Percent of Stores Stocking Any Food in All 10 Categories; T1 = 2014 (Pre-policy), T2 = 2015 (Implementation only), T3 = 2016 (Early initiation of enforcement), T4 = 2017 (Continued monitoring)

**Fig. 4:**
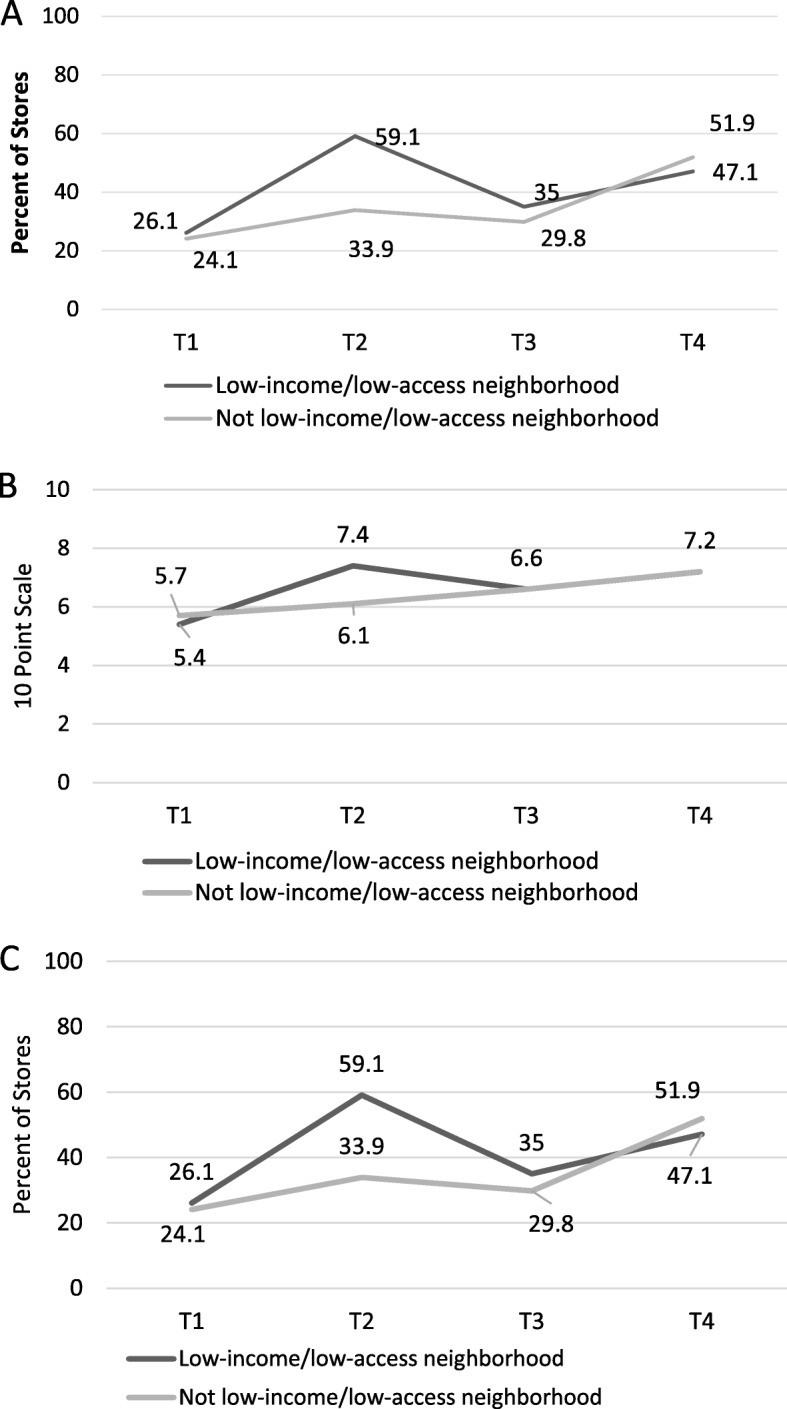
Compliance with the Minneapolis Staple Foods Ordinance in Lower-Income/Access and Higher-Income/Access Stores in Minneapolis, MN **A**: Percent of Stores Meeting 80% of Staple Foods Ordinance Standards; **B**: 10-Point Scale of Meeting Ordinance Requirements; **C**: Percent of Stores Stocking Any Food in All 10 Categories; T1 = 2014 (Pre-policy), T2 = 2015 (Implementation only), T3 = 2016 (Early initiation of enforcement), T4 = 2017 (Continued monitoring)

Table 4 presents the results of models and significance testing for compliance indicators in Minneapolis by neighborhood factors; Figs. 3 and 4 visually present trends in compliance by neighborhood over time. Changes in stores in higher-SES areas were generally similar to changes in lower-SES areas, with no statistically significant differences. In low-income/low-access areas compared with areas that were not low-income/low-access, the magnitude of changes in compliance between T1 and T4 was similar for all outcomes. Outcomes for stores within vs. outside low-income/low-access were similar at T1, and also similar at T4, though overall trends were different for the 10-point scale (*p* = 0.002) and carrying any items in each of the 10 categories (*p* = 0.05). Adjusting for store factors (size and ownership status) did not result in substantive changes in neighborhood trends.

**Table 4:** Compliance by neighborhood characteristics (Minneapolis only)

Compliance Outcome	Neighborhood Characteristic	Pre-policy (2014) Mean (SE)	Implementation only (2015) Mean (SE)	Early initiation of enforcement (2016) Mean (SE)	Continued monitoring (2017) Mean (SE)	Overall Effect (time-by-characteristic) *p* (df = 3)
Met at least 8 of 10 ordinance standards (% of stores)	Lower SES	26.9 (8.7)	48.2 (9.6)	42.3 (9.7)	44.0 (9.9)	0.23
	Higher SES	23.5 (5.9)	37.3 (6.8)	25.5 (6.1)	54.4 (7.3)	
	Low-income/low-access	26.1 (9.2)	59.1 (10.5)	35.0 (10.7)	47.1 (12.1)	0.15
	Not low-income/low-access	24.1 (5.8)	33.9 (6.3)	29.8 (6.1)	51.9 (6.8)	
10 point scale (0–10; 1 point for each category where standard met)	Lower SES	5.9 (0.5)	6.9 (0.5)	6.8 (0.4)	6.7 (0.4)	0.12
	Higher SES	5.5 (0.4)	6.3 (0.3)	6.5 (0.3)	7.4 (0.3)	
	Low-income/low-access	5.4 (0.6)	7.4 (0.5)	6.6 (0.6)	7.2 (0.5)	0.002
	Not low-income/low-access	5.7 (0.3)	6.1 (0.3)	6.6 (0.3)	7.2 (0.3)	
	*p*-net		0.001	0.65	0.70	
Any food in each of 10 categories (% of stores)	Lower SES	30.8 (9.1)	44.4 (9.6)	57.7 (9.7)	72.0 (9.0)	0.82
	Higher SES	25.5 (6.1)	35.3 (6.7)	47.1 (7.0)	76.1 (6.3)	
	Low-income/low-access	30.4 (9.6)	45.5 (10.6)	40.0 (11.0)	82.4 (9.2)	0.05
	Not low-income/low-access	25.9 (6.0)	35.7 (6.4)	54.4 (6.6)	72.2 (6.1)	
	*p*-net		0.73	0.09	0.65	

Bolded entries: *p* < 0.05; Low SES: > 50% of residents at 185% of poverty; High SES: ≤50% of residents at 185% of poverty; All models adjusted for repeated measures over time; *p*-net values refer to changes in time-by-characteristic effect from pre-policy to implementation only, from pre-policy to early initiation of enforcement, from pre-policy to continued monitoring respectively

## Discussion

Previous work indicated that compliance with the Minneapolis Staple Foods Ordinance was low, with only 10% of small and non-traditional Minneapolis food retailers meeting full compliance; moreover, Minneapolis was no different from St. Paul in terms of change in full retailer compliance across the study period [26]. The current study adds to that work, with the objective of assessing three indicators of movement towards compliance, and assessing compliance in different stores and neighborhoods. The current study shows that stores in Minneapolis demonstrated robust increases in compliance measures in the 3-year period following ordinance implementation. Minneapolis stores had a greater magnitude increase in carrying any items in all 10 product categories of the ordinance compared with St. Paul stores. In addition, corporate stores may be better equipped to make changes to increase compliance than independent stores, and that there were not meaningful differences in change in compliance between neighborhood types. These findings do not diminish the implementation concerns cited in the previous study, but they do demonstrate movement towards the goal of the ordinance to provide a minimum level of healthy food in stores across the city. However, it is possible that changes observed are attributable to factors other than the ordinance, such as secular trends in the marketplace, changes in food preferences of shoppers, and/or changing demographics of shoppers.

Results from different compliance measures offer some insight into the successes and challenges of the ordinance. Gains in carrying any food in each of the 10 required categories indicate stores’ ability to obtain different types of healthy, staple foods. This includes obtaining a supplier or the capacity to self-supply certain types of food, as well as having the basic infrastructure for stocking perishable products. Indeed, capacity to supply and infrastructure to stock a variety of healthy foods has consistently been cited in previous literature as central to the success of small stores in carrying healthy products [7, 8]. Increases in carrying food in each of the 10 categories suggest a promising step in compliance; the statistically significant increase in Minneapolis compared with St. Paul stores suggest that Minneapolis stores may have expanded their network of suppliers and/or capacity to self-supply healthy and staple foods. Stores that carry any food in each of the 10 categories, but do not meet full ordinance requirements, may stock less healthful versions of required foods (e.g., whole/reduced-fat milk instead of low-fat/skim milk) or narrowly miss ordinance requirements while still carrying healthful products (e.g., eggs in ½-dozen containers instead of 1-dozen containers). For such stores, it remains unclear whether the lack of full compliance is due to insufficient retailer understanding about the ordinance, inadequate space to stock required amounts, manager beliefs about the demand for certain products, or a combination of these and other factors.

Corporate stores demonstrated greater gains in compliance during the study period compared with independent stores, respectively. Corporate stores scored considerably lower on all measures at baseline, but had surpassed independent stores on all measures by T4. Corporate stores may be better positioned to bring themselves into alignment with the policy due to economies of scale in purchasing and delivery. Additionally, corporate stores’ centralized decision-making could have facilitated compliance across many stores at once. Given their prominence in the food retail landscape, corporate stores may have had more pressure to comply from those in charge of ordinance implementation. Larger stores may share some characteristics with corporate stores that make it easier to supply healthy food (e.g., economies of scale); indeed, larger stores demonstrated a greater magnitude of change than smaller stores, although these changes were not always statistically significant.

Compliance trends were similar for stores in lower-SES and higher-SES neighborhoods. Progress was more variable over time for stores within versus outside low-income/low-access areas, and increases were not observed at every study time point. However, by T4, total increases in compliance were similar for stores in low-income/low-access areas compared to those outside low-income/low-access areas. Overall, findings do not raise concerns about differences in compliance across these neighborhood characteristics.

In 2018, after our data were collected, concerns were raised about insufficient flexibility of stocking options in the Staple Foods Ordinance, and in December 2018 Minneapolis City Council approved modifications to the ordinance requirements that improved flexibility in providing healthy and culturally-appropriate food that meets customer demand [36]. As of early 2019, the Minneapolis Health Department was continuing to offer education, training, and technical assistance to help assist with compliance, albeit with increasingly limited by budgetary cuts for health inspections. Low compliance with the ordinance remains a concern, despite overall trends suggesting broad movement of Minneapolis stores towards providing a minimum level of healthy food. Meanwhile, there is national interest in minimum stocking requirements policies for improving healthy food in small food retailers. In 2016, the Robert Wood Johnson Foundation published a set of recommendations for healthy stocking criteria for small retail food stores meant to inform similar initiatives [37], and another city recently passed a similar Staple Foods policy [38]. Other cities are actively considering the feasibility of similar policies.

This study has a number of strengths, including hypotheses grounded in a health disparities framework, a well-matched comparison condition, and four time points of objective measurement of small and non-traditional food stores that spanned the period of pre- and post-policy implementation. One limitation of the study is that changes in two of our three indicators did not differ significantly by city, so compliance changes in Minneapolis may be attributable to factors other than the ordinance. Another limitation is that our analysis did not test compliance mediators; while we can glean from previous studies what challenges may have been likely for different kinds of stores [7–9], these were not tested in the current study. Another limitation is the sample size for the Minneapolis-only analyses, which may not have been large enough to detect significant subgroup differences; our conclusions are supported by both visual trends and *p*-values. Finally, the study observed only one geographic area and enforcement of the ordinance was limited; thus, there may be other circumstances under which to test the feasibility of implementing minimum stocking requirement policies [8].

## Conclusions

The experience of Minneapolis in implementing the first Staple Foods Ordinance suggests that the success of implementing minimum stocking requirements in small/non-tradition retailers may depend on store factors related to store capacity, such as ownership status. However, location differences in compliance were not evident, suggesting that the policy did not exacerbate urban food access disparities. Given national interest in minimum stocking requirements for small food stores, additional work is needed to understand implementation challenges in different retail settings.

## Data Availability

The datasets generated during the current study are not publicly available because the investigator team is still actively analyzing and publishing study results, but data are available from the corresponding author on reasonable request. We will reassess making.the data publicly available when current analyses are completed.

## Abbreviations

ACS: American Community Survey estimates
MN: Minnesota
NEMS-S: Nutrition Environment Measure Survey in Stores
SES: Socioeconomic status
SNAP: Supplemental Nutrition Assistance Program
USDA: U.S. Department of Agriculture
WIC: Special Supplemental Nutrition Program for Women, Infants and Children
